# Rapid Leaf Deployment Strategies in a Deciduous Savanna

**DOI:** 10.1371/journal.pone.0157833

**Published:** 2016-06-16

**Authors:** Edmund Carl February, Steven Ian Higgins

**Affiliations:** 1 Department of Biological Sciences, University of Cape Town, Private Bag, Rondebosch, 7701, South Africa; 2 Department of Botany, University of Otago, PO Box 56, Dunedin 9054, New Zealand; 3 Biodiversity and Climate Research Centre (BiK-F), Senckenberg Gesellschaft für Naturforschung, Senckenberganlage 25, 60325, Frankfurt am Main, Germany; DOE Pacific Northwest National Laboratory, UNITED STATES

## Abstract

Deciduous plants avoid the costs of maintaining leaves in the unfavourable season, but carry the costs of constructing new leaves every year. Deciduousness is therefore expected in ecological situations with pronounced seasonality and low costs of leaf construction. In our study system, a seasonally dry tropical savanna, many trees are deciduous, suggesting that leaf construction costs must be low. Previous studies have, however, shown that nitrogen is limiting in this system, suggesting that leaf construction costs are high. Here we examine this conundrum using a time series of soil moisture availability, leaf phenology and nitrogen distribution in the tree canopy to illustrate how trees resorb nitrogen before leaf abscission and use stored reserves of nitrogen and carbon to construct new leaves at the onset of the growing season. Our results show that trees deployed leaves shortly before and in anticipation of the first rains with its associated pulse of nitrogen mineralisation. Our results also show that trees rapidly constructed a full canopy of leaves within two weeks of the first rains. We detected an increase in leaf nitrogen content that corresponded with the first rains and with the movement of nitrogen to more distal branches, suggesting that stored nitrogen reserves are used to construct leaves. Furthermore the stable carbon isotope ratios (δ^13^C) of these leaves suggest the use of stored carbon for leaf construction. Our findings suggest that the early deployment of leaves using stored nitrogen and carbon reserves is a strategy that is integrally linked with the onset of the first rains. This strategy may confer a competitive advantage over species that deploy leaves at or after the onset of the rains.

## Introduction

Savanna is characterised by a discontinuous mosaic of trees with a grass understory [1]. Coupled with high diurnal temperatures, rainfall in savanna is distinctly seasonal with very pronounced wet and dry cycles. During the dry season rainfall may be extremely low or entirely absent for a few to several weeks. As a result, soil water availability is recognised as one of the key factors constraining maximum woody cover at more arid sites, whereas at less arid sites it is proposed that woody cover is not limited by moisture but by fire and herbivory [2, 3]. Research in the Kruger National Park in South Africa using stable water isotope ratios has shown that although riparian evergreen trees such as *Philenoptera violaceae* use deep water deciduous savanna trees such as *Colophospermum mopane* are reliant on regular rainfall [4]. This reliance on rainfall, combined with the rainfall seasonality, effects a decline in soil moisture in the dry season resulting in a suspension of growth in trees. In many tree species the dry season suspension of growth is additionally associated with deciduousness [5–8].

Several factors have been hypothesised to promote deciduousness in seasonally dry environments. For example, deciduous trees do not have to carry the costs of leaf respiration or contend with herbivory in the dry season [9, 10]. The liability is that deciduous trees have to construct new leaves, which is expensive both in nitrogen and carbon. The leaves of deciduous trees typically have higher nitrogen content, photosynthesis and specific leaf area than those of evergreen trees. This allows them to fix sufficient amounts of carbon in the relatively short growing period [9–11]. Several recent studies have suggested that nitrogen is a limiting resource in both South African and South American savannas [12–14]. It has also been shown that nitrogen is highest in the top 10 cm of the soil and that the majority of roots are also in this layer of the soil [15, 16], that is plants in these systems allocate their roots preferentially in areas where nitrogen concentrations are highest. Furthermore, this nitrogen is available only briefly to plants, supplied as a pulse at the start of the growing season. [17–19].

The high nitrogen demands of deciduousness, the low average nitrogen availability, and the seasonal pattern of nitrogen and water availability in the study system imply that nitrogen conservation may be important. Deciduous trees can conserve nitrogen through nutrient resorption or the withdrawal of nutrients from senescing leaves and the storage of these nutrients in woody parts [20–23]. Many savanna trees deploy new leaves, just before the rains, using these stored resources [24–26]. All deciduous trees at our study site may flush either before or with the first rains. Some species such as *Sclerocarya birrea* flush well before the rains (early flushing) while others such as *Combretum apiculatum* flush shortly before or with the first rains (late flushing) [6]. While the early flushing trees clearly pre-empt rainfall the late flushing trees do so to a lesser extent [6]. In this study we examine whether trees resorb nitrogen from leaves at the end of the growing season and whether stored carbon and nitrogen resources are used to deploy new leaves at the onset of the growing season. We do this to elucidate how trees can afford deciduousness in a nitrogen limited ecosystem.

## Methods

### Declaration

The study did not involve any humans or animals and permission to do the research in the Kruger National Park was granted by South African National Parks Scientific Services in the Kruger National Park. Our data are available on the national park web site at; http://dataknp.sanparks.org/sanparks/metacat/judithk.110818.7/sanparks

### Study Site

The study site is located in the southern and south western part of the Kruger National Park, Mpumalanga Province, South Africa. Rainfall for the region is distinctly seasonal with hot, wet summers and cool, dry winters. The growing season starts in October and ends in April. Many trees are deciduous and grasses inactive in the dry season. Mean annual maximum and minimum temperatures at Skukuza (24°59'44.88"S 31°35'30.84"E) for the 30 years from 1981 to 2010 was 29.9°C and 14.7°C with mean annual precipitation 590 mm (South African Weather Service. WB42, Climate Statistics). Temperatures range between a maximum of 32.6°C in January to a minimum of 6.2°C in June.

The vegetation for the area is classified as granite lowveld by Mucina and Rutherford [27] with *Sclerocarya birrea* (A.Rich) Hochst, *Combretum apiculatum* (Sond.), and *Terminalia sericea* (Burch. Ex DC), common trees. Grasses are dominated by *Hyperthelia dissoluta* (Nees ex Steud) Clayton and *Elionurus argenteus* Nees. Soil depth is variable but these nutrient poor granite soils are seldom deeper than 1.5 m to bedrock in our study area [15, 28]. The basement geology for the area is comprised of granite, gneiss and migmatite (Nelspruit granite suite, [29]). These basement rocks weather into distinct catenal sequences with sandy soils on the uplands and clayey soils in the bottomlands [27]. Soil nitrogen for the region is 0.52 mg g^-1^ soil and phosphorous (Bray II) is 3.23 mg kg^-1^ soil [12].

### Rainfall, Soil moisture and Leaf Phenology

Weather stations were located at (1) 25°4'21.71"S 31°32'48.50"E and approximately 5 kms from site 1, (2) 25°6'39.60"S 31°30'54.00"E and approximately 14 kms from site 2, (3) 25°5'27.60"S 31°22'51.60"E. These weather stations included a Decagon Devices EC-5 soil moisture probe (Pullman, WA, USA) which was inserted 5 m away from the weather station horizontally into the soil at a depth of 20 cm while rainfall and temperature were recorded using a Rain-O-Matic tipping bucket rain gauge (Pronamic, Ringkøping, Denmark) and a Vaisala HMP50 temperature sensor (Campbell Scientific, Logan, UT, USA). These instruments were controlled with a CR200 data logger (Campbell Scientific Inc., Logan, UT, USA), which was powered by a rechargeable lead acid battery connected to a voltage-regulated solar panel. For rainfall and soil moisture we average the values for the stations at our three study sites.

At each of these three study sites we also tagged three *Terminalia sericea* and three *Combretum apiculatum* trees. For each individual tree, an index of leaf canopy fullness was estimated [8]. Two observers scored the percentage of leaves relative to the potential canopy area of leaves for each study tree every second week from 6^th^ January 2009 to 6^th^ January 2010. Scores were as follows: 0 represented no leaves, 1<2%, 2 between 2% and 25%, 3 between 26% and 50%, 4 between 51% and 75% and 5 >75% of the total canopy. The number reached is firstly an independent estimate and then a consensus between the two observers as to an appropriate value for each tree. This consensus score was recorded.

### Nutrient resorption

For each study tree described in the previous section we measured how nitrogen content changed in leaves and in twigs of different branch orders as the growing season initiated and terminated. We randomly collected ten fully expanded fresh new leaves from each of these study individuals at the end of each month at the end (March to June 2009) and beginning (Oct, Nov, Dec 2009) of the growing season. At the same time we also collected a 4 cm long twig sample from each of the first three branching orders using a pair of secateurs. We used a power drill to collect the sample from the 4^th^ branching order. We denoted these branching orders analogous to how stream orders are denoted; order-one being the terminal branch, order two being the point below where two order-one branches join, order-three being where two order-two branches join and so on.

Both leaf and wood samples were dried to a constant weight at 70°C in a forced convection oven (Scientific, series 200 oven, South Africa) and the bark and pith removed from the twig before grinding to a fine powder using a Retsch MM200 ball bearing mill (Retsch, Haan, Germany). Stable carbon isotope ratios relative to PDB (δ^13^C) and percentage nitrogen were then determined on these samples using a Thermo Finnigan Delta plus XP Mass Spectrometer coupled to a Thermo Finnigan Flash EA1112 Elemental Analyser with automatic sampler (Thermo Electron Corporation, Milan Italy).

### Statistical analysis

The change in leaf nitrogen content was analysed as a linear regression against time (in months from the start of the growing season). The twig nitrogen content (N) are analysed using a linear model,

$$N\sim slope1\left[order\right]\times time+slope2\left[order\right]\times time^{2}+intercept\left[order\right]+tree$$

In this model time counts from 3 to 12 (March to December) and the quadratic term is used to describe the non-linearity of how nitrogen changes with time for some twig orders. Separate slope and intercept parameters are estimated for each twig order. Tree is treated as a random effect. The parameters of the model (S1 Code Listing) were estimated using JAGS [30]. JAGS is a general purpose software for fitting hierarchical Bayesian models using Markov Chain Monte Carlo (MCMC) simulation. The response variable was assumed to be normally distributed and un-informed priors were used.

## Results

### Rainfall Soil moisture and Leaf Phenology

**Fig 1:**
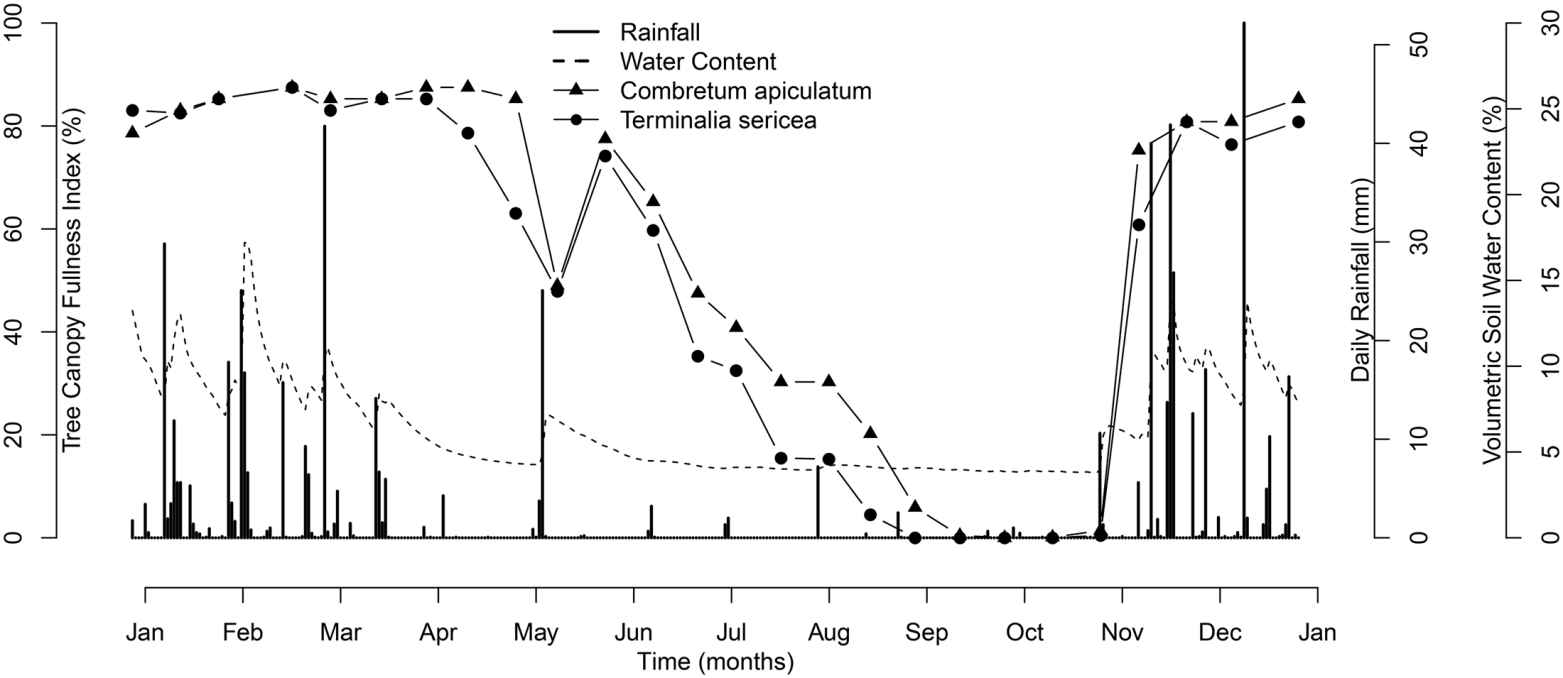
Time series of daily rainfall, volumetric soil water content and tree canopy fullness index for *Combretum apiculatum* and *Terminalia sericea*.

As rainfall decreased from April to October (Fig 1) both tree species gradually lost their leaves. For *T*. *sericea* this reduction in leaf canopy area was faster than for *C*. *apiculatum* but by the middle of October 2009 both species had lost all their leaves (Fig 1). As soil moisture increased with the first rainfall at the beginning of November both species exhibited an immediate and rapid increase in leaf area. There were no leaves on our *T*. *sericea* study trees on the 21^st^ of October and only a few leaves on two of our *C*.*apiculatum*, (0 mm of rain in the preceding 2 weeks) on the 5^th^ of November (12.4 mm of rain) there were a few leaves on all trees and by 17^th^ November (48 mm of rain) between 50 and 75% of total potential canopy (Fig 1). Rainfall at our study site from the 1 st September to 21^st^ October was 2.3 mm ranging in amount from 0.1 mm to 1 mm. Mean maximum temperature for this time was 30°C while minimum temperatures were 15°C for September and 17°C for October.

### Nutrient resorption

**Fig 2:**
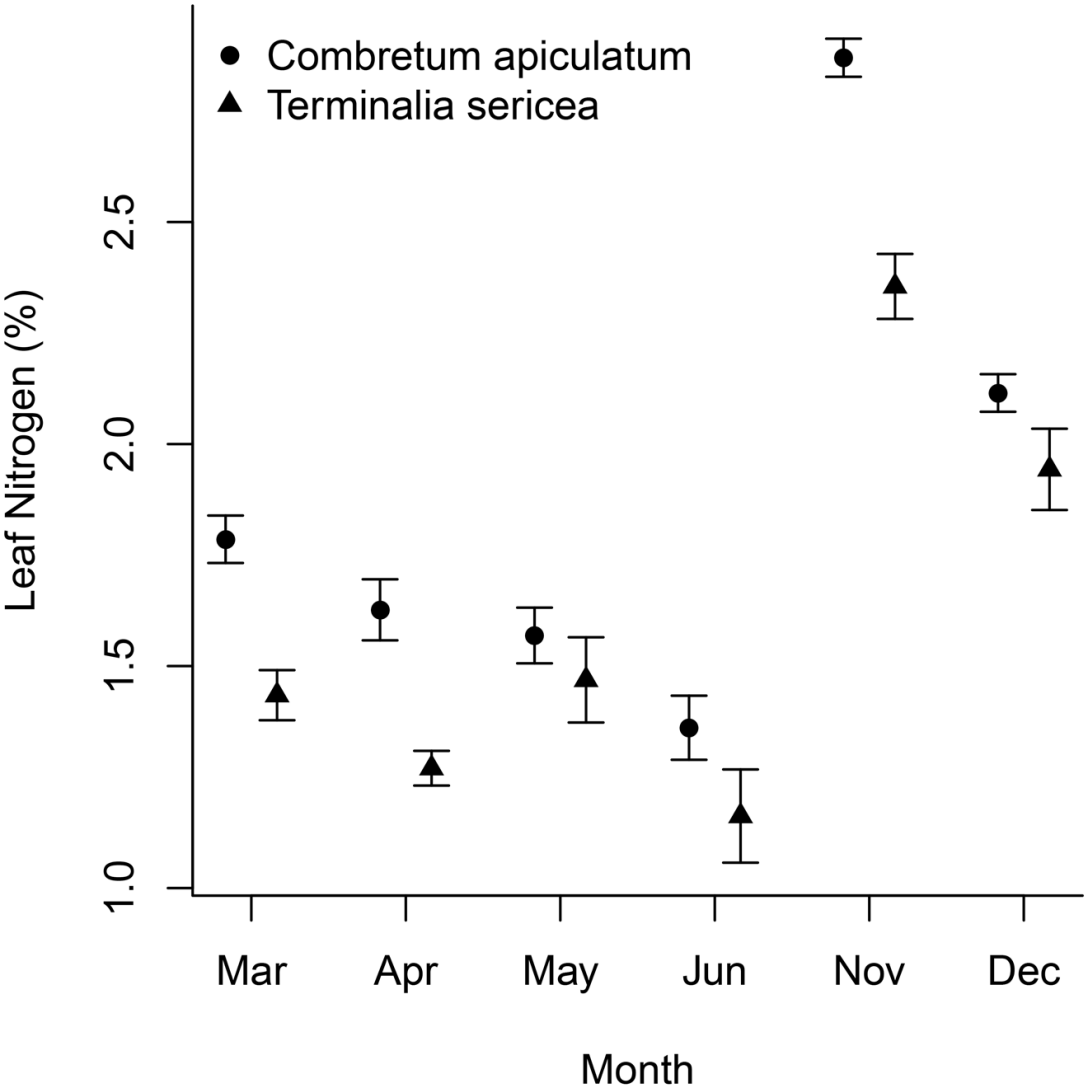
The percentage leaf nitrogen per unit dry mass against time since the start of the growing season (November to June) for *Combretum apiculatum* and *Terminalia sericea*. No estimate was available for October because no leaves were present. Grey bars are *T*. *sericea* and open bars *C*. *apiculatum*. Values are means ± 1 SE.

For both *T*. *sericea* and *C*. *apiculatum* leaf percentage nitrogen decreased linearly with time from the beginning of the wet season (Nov, Dec) through to the middle of the dry season in June (Fig 2, Table 1). The rate of this decrease was similar for the two species: the 95% credible intervals of the slopes of these regressions spanned -0.21 to -0.16 for *C*. *apiculatum* and -0.18 to -0.12 for *T*. *sericea* (Table 1). The intercepts were higher for *C*. *apiculatum* than for *T*. *sericea* indicating that *C*. *apiculatum* had higher leaf nitrogen contents.

**Table 1:** Slope (% nitrogen per month) and intercept (% nitrogen) of a linear regression of leaf nitrogen content against time since the start of the growing season (November to June) for *Combretum apiculatum* and *Terminalia sericea*.

	Slope (95% quantiles)	Intercept (95% quantiles)
***C*. *apiculatum***	-0.179 (-0.21;-0.15)	2.742 (2.58;2.91)
***T*. *sericea***	-0.152(-0.18;-0.12)	2.339 (2.16; 2.51)

**Fig 3:**
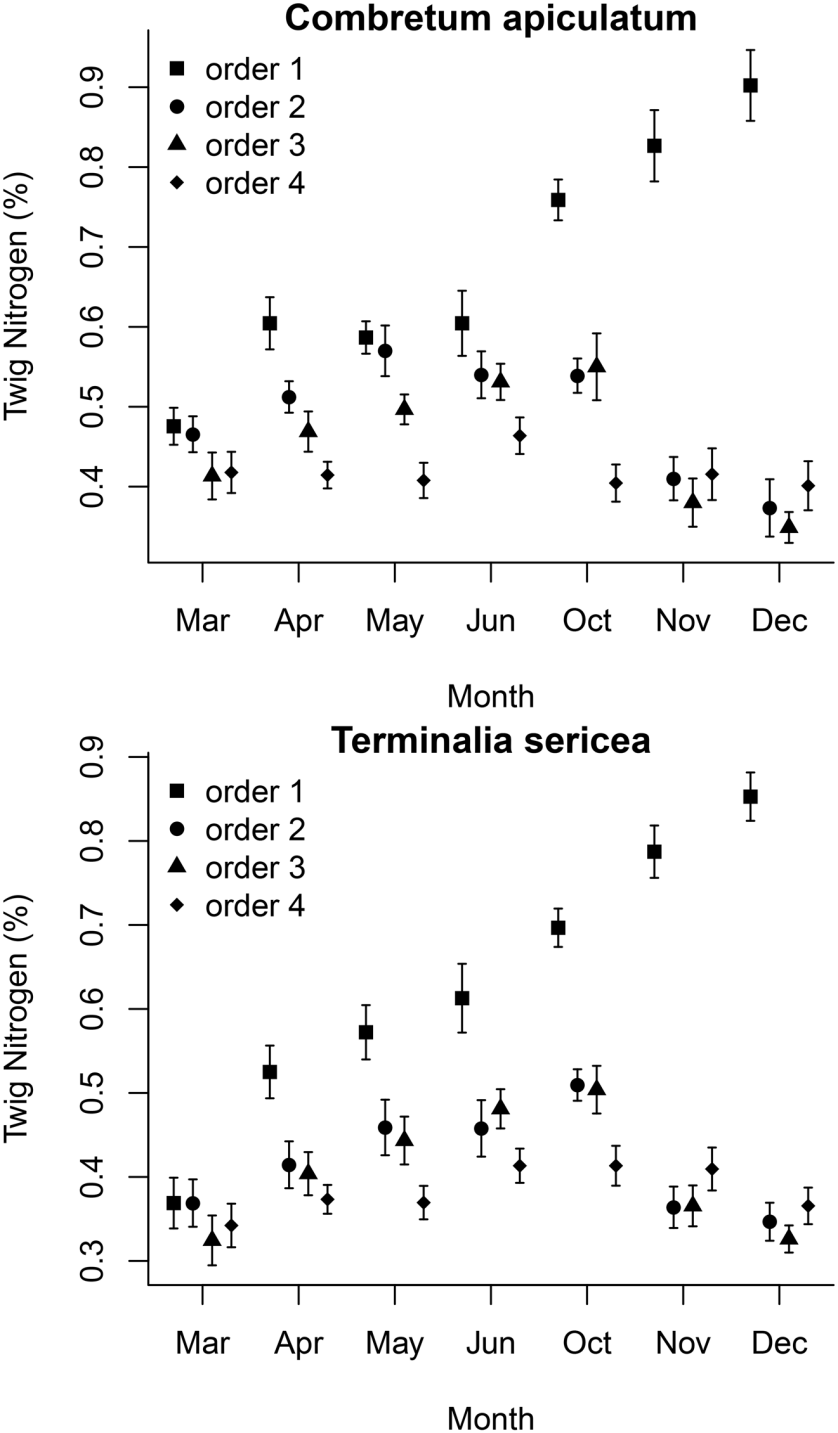
Seasonal variation of nitrogen content for twigs of different branching orders (order 1 terminal, order 4 more basal) of *T*. *sericea* (top) and *C*. *apiculatum* (bottom). Values are means ± 1 SE.

**Fig 4:**
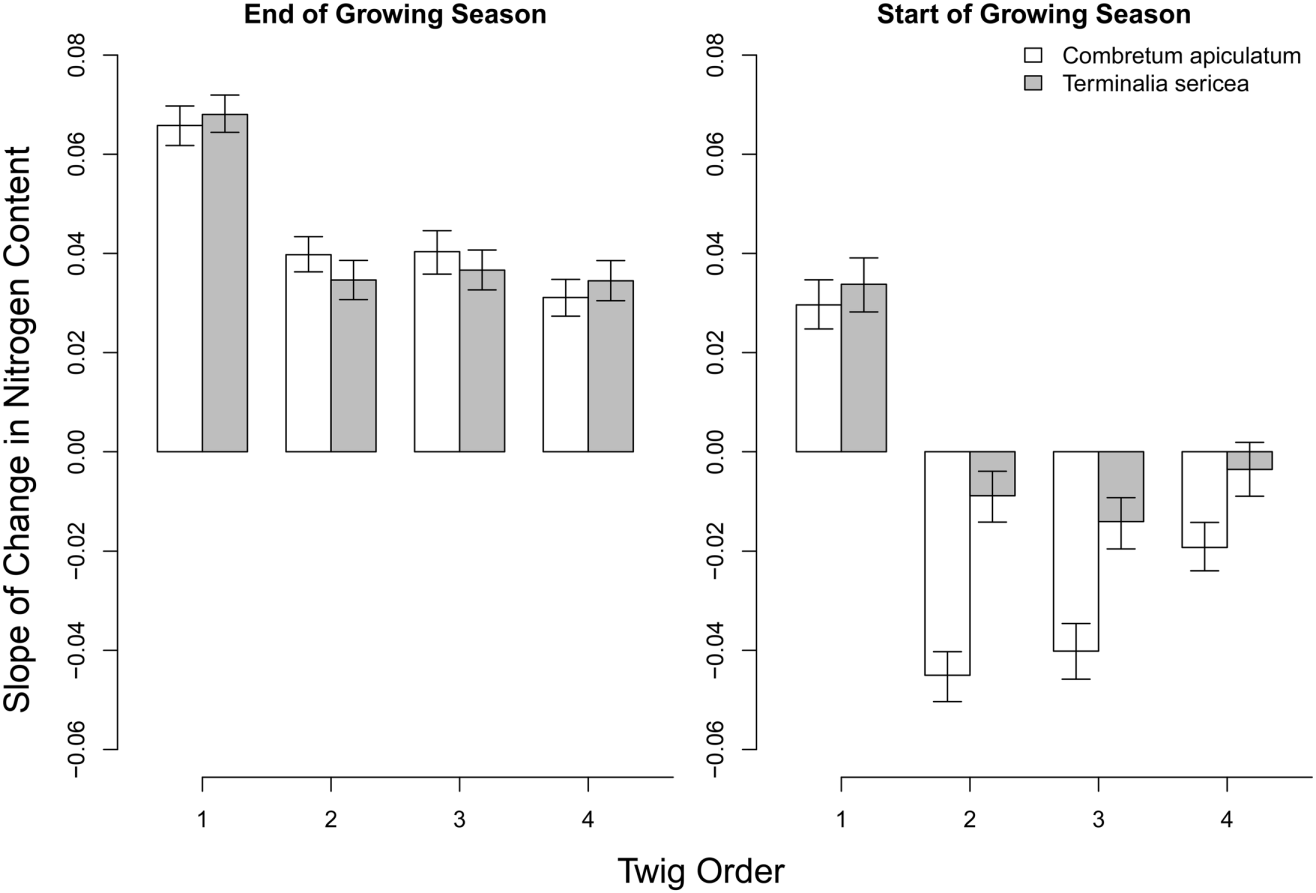
Slope of the relationship between twig nitrogen content and time at the end (March to April) and start (November to December) of the growing season for *C*. *apiculatum* and *T*. *sericea*. The bars indicate the estimated slopes and the error bars indicate the 95% credible intervals of the estimates.

**Fig 5:**
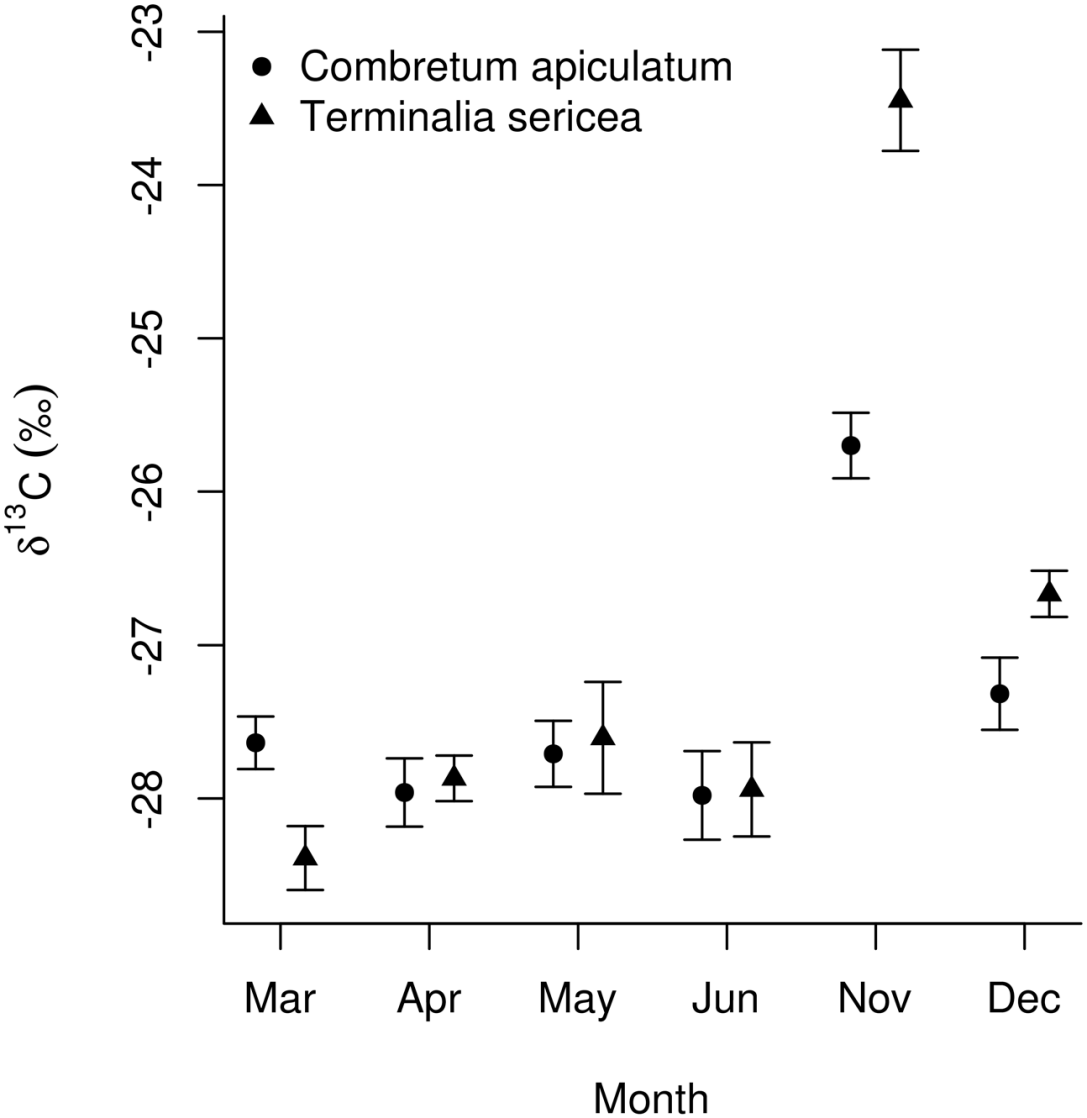
Leaf δ^13^C values against time since the start of the growing season (November to June) showing the relatively enriched ^13^C values in November relative to other times of the year. Values are means ± 1 SE.

At the end the growing season (March to April) nitrogen content increased in all branch orders (orders 1 to 4), consistent with resorption of nitrogen into deeper branch orders (Figs 3 and 4). At the start of the growing season (November to December) nitrogen content decreased in deeper (orders 2,3,4) branch orders, but increased in branch order 1; changes that are consistent with a movement of nitrogen from basal twigs to terminal twigs (Figs 3 and 4). All change slopes reported differed from zero except for the changes in the deeper branch orders at the start of the growing season for *T*. *sericea*. The slopes shown in Fig 4 were derived from the fitted polynomial models and their full conditional posterior distributions were computed (S1 Code Listing). Concomitant with this, the first fully expanded leaves of the growing season (November) have δ^13^C values that are enriched relative to any other month of the year (P<0.001). For *C*. *apiculatum* these values are -25.7 ± 0.23‰ for November with a range from-27.3 ± 0.2 to -28.0 ± 0.3‰ for the rest of the year (Fig 5). For *T*. *sericea* values are -23.4 ± 0.3‰ for November with a range from -26.6 ± 0.1‰ to -28.4 ± 0.2‰ for the rest of the year (Fig 5).

## Discussion

The study system has a dry and cool unfavourable season and a wet, warm growing season resulting in a strong seasonality in leaf phenology [11, 25]. Our study species were completely deciduous, with no leaves on the trees at the end of the dry season. The patterns of canopy leaf fullness were correlated with rainfall, for example both species show an increase in canopy volume in response to a late rainfall event in May. While leaf fall may be closely aligned with rainfall amount several studies have suggested that leaf emergence may be decoupled from soil moisture [6, 11, 25]. Using a multi-spectral camera to track the normalized difference vegetation index (NDVI) of individual canopies Higgins et al [6] detected that leaf emergence pre-empted rainfall at the same study site. Although the Higgins et al [6] study identified individual tree canopies they could not assign species names to these canopies. Our results however show that only two of our study *C*. *apiculatum* trees had some leaves before the rains while there were no leaves on the *T*. *sericea* until after the rainfall event of the 30^th^ October. This rain event at the end of October triggered bud-break and rapid leaf growth as we observed an extremely rapid expansion of the canopy for both species in response to the first rains from a few leaves on the trees at the end of October to a full canopy within two weeks of the first rains. This canopy response coincides with the flush of plant available nitrogen that becomes accessible when the soil is rewetted with the first rains at the end of the dry season [17–19].

The new leaves produced at the beginning of the growing season were enriched in the heavier ^13^C isotope relative to other times of the year. Several studies have shown that leaves derived from stored reserves have δ^13^C values that are more enriched than leaves derived from recent photosynthesis [31, 32]. The reason for this is that part of these reserves is comprised of non-structural carbohydrates which have a more enriched (up to 4 ‰) δ^13^C value than those derived from recent photosynthesis [33, 34]. The depletion we observed in leaf δ^13^C values as the growing season progressed, for both of our study species, is consistent with the carbohydrates in these leaves being replaced by assimilates derived from more recent photosynthesis [32]. These results suggest that our study trees are using stored carbon reserves in the construction of new leaves.

In low nutrient environments such as at our study site trees may resorb nutrients from the leaves prior to abscission [13, 22]. Such a strategy divorces a plant from a reliance on a continuous and current nutrient supply. Our results show that nitrogen is transported from the leaves to the more proximal branches as leaf abscission progresses. At the onset of the next growing season this nitrogen is then remobilized to more terminal branches. This mobilisation of stored nitrogen and carbon allowed our study species to deploy new leaves and construct a full leaf canopy within two weeks of the first rainfall event. We therefore propose that trees in our study system can afford the cost of deciduousness by withdrawing and conserving both nitrogen and carbon.

In this study we use the two most common tree species at our study site and found that both follow the same strategy. That is, both species flush their leaves early synchronous with rainfall and are able to rapidly construct a full canopy after that. It may be argued that the primary function of rapid leaf deployment is to satiate browsing herbivores, which may be particularly beneficial at the beginning of the growing season when browse availability is limited [35]. If this were true selection might favour cheating where individual trees that did not invest in early-deployment would deploy leaves later once herbivores are satiated. An alternative is that the rapid construction of a full canopy of leaves allows plants to set-up transpiration gradients that, through mass flow from the soil to the roots, maximises uptake when nitrogen becomes available in a pulse at the beginning of the growing season [13, 36, 37]. In a previous study at this site we detected a large pulse of nitrogen with the first rains [19] and this relationship between rainfall and nitrogen mineralization has also been demonstrated in a bimodal rainfall savanna in Senegal [38]. The rapid construction of a full canopy of newly developing leaves may additionally set-up source sink gradients to initiate root development [39, 40].

Our findings suggest that the deciduous strategy of savanna trees is tightly linked to the seasonal pattern of nitrogen availability and that early deployment of leaves using stored nitrogen and carbon reserves is integral to this strategy. Future studies should examine whether the strategy of rapid leaf deployment allows trees to compete more effectively for soil nitrogen than a later flushing strategy.

## Supporting Information

S1 Code Listing JAGS Code listing for the statistical model used to generate the parameters shown in the main manuscript Fig 4. "
(BUG)
